# Editorial: Advances in Emotion Regulation: From Neuroscience to Psychotherapy

**DOI:** 10.3389/fpsyg.2017.00985

**Published:** 2017-06-21

**Authors:** Alessandro Grecucci, Jon Frederickson, Remo Job

**Affiliations:** ^1^Clinical and Affective Neuroscience Lab, Department of Psychology and Cognitive Sciences, University of TrentoRovereto, Italy; ^2^Washington School of PsychiatryWashington, DC, United States; ^3^ISTDP InstituteWashington, DC, United States

**Keywords:** emotion regulation, psychotherapy, affective neuroscience, memory reconsolidation

## Overview

Emotions are the gift nature gave us to help us connect with others. Emotions do not come from out of nowhere. Rather, they are constantly generated, usually by stimuli in our interpersonal world. They bond us to others, guide us in navigating our social interactions, and help us care for each other. We love our partner, we get angry with a friend, we feel sad for the loss of a parent, and so on…Paraphrasing Shakespeare “*Our relationships* are such stuff as *emotions* are *made* of.” Within our relationships, emotions express our needs and desires. When problems happen in our relationships, emotions arise to help us fixing such problems (Frederickson, 2013). However, sometimes emotions can become dysregulated and pathology begin. It is now widely accepted that almost all forms of psychopathologies are associated with specific dysregulated emotions or dysregulatory mechanisms (Grecucci et al., 2016a). If it is true that problems occur within relationships, it is also true that *the solution occurs there*. We are hurt *in a relationship*, and we are healed *in a relationship*. That is why and how psychotherapy works. Emotions that becomes dysregulated inside our relationships, can become regulated in an *ad hoc designed therapeutic relationship* where the therapist helps the client to express, face and regulate her/his emotions, and channel them into healthy actions. The idea behind this research topic is to gather contributions for the first time from both affective neuroscientists and psychotherapists to shed light on the ways our emotions become dysregulated in life and can become regulated through psychotherapy. We present novel approaches and strategies to regulate emotions that are strongly grounded in affective neuroscience and experimental research. We strongly believe it is time that researchers in affective science and clinicians make a collective effort to deepen the understanding and the practice of how emotions can be usefully elaborated in clinical settings. The Topic is divided in two sections, the first more experimental and the second more clinical.

## Part 1: advances in the neurocognitive mechanisms of emotion regulation and dysregulation

The first section of the special issue starts with a reflection on the importance of distinguishing *explicit* emotion regulation based on conscious and effortful application of strategies from *implicit* emotion regulation based on automatically and unconsciously designed mechanisms (Rice). Parallels are made with the psychoanalytic concept of defense mechanisms as a form of implicit emotion regulation. Another aspect explored in this part is the role of empathy in mediating the association between difficulties in emotion regulation and hostility (Contardi et al.). Cai et al. explore how sex and extraversion modulate self-reported emotional experience in an ERP experiment. The authors suggest that there is a male advantage for using expressive suppression for emotion regulation in non-extraverted, ambivert individuals. Deficits in the regulation of interpersonal emotions have been linked to severe psychiatric disorders. Understanding how patients experience and fail to regulate such emotions is of fundamental importance (Grecucci et al., 2015a). Depression is strongly characterized by difficulties in regulating unpleasant emotions. An intriguing psychodynamic hypothesis considers depression as a failure in mother-infant interactions during childhood that affects the construction of the representation of the self, others, and relationships. Messina et al. provide a link between abnormal activation of the default system in the brain observed in depression and the exaggerated negative self-focus and rumination that lead to emotion dysregulation in these patients. Clinical implications are also discussed.

Individuals with marked autistic traits display several features of social and emotion dysregulation. Imageability ratings of word classes denoting proprioceptive, emotional, and theoretical words predict whether people have low or high autistic traits, or, to put it differently, whether they have more or less marked empathic inclinations (Esposito et al.). People with anxiety disorders suffer from severe emotion dysregulation and subsequent cognitive biases. Cui et al. explore how the emotional context affects successful and unsuccessful source retrieval amongst high-trait-anxiety college students by using event-related potentials (ERPs).

## Part 2: new therapeutic protocols to foster emotion regulation in psychotherapy

The ability to regulate emotions is essential for healthy psychological functioning and is a key focus of psychotherapy. Working actively with emotion has been empirically shown to be of central importance in psychotherapy. Different therapeutic models from different theoretical orientations have incorporated principles and techniques to work on dysregulated emotions. In this section, we present novel models of treatments to regulate emotions that therapists can use in clinical practice. We start by presenting Emotion Regulation Therapy (ERT) (Renna et al.), an evidence-based treatment that integrates contemporary psychotherapy modalities with basic and translational affective science to offer a framework for improving emotion regulation in patients. Strategies, technique and clinical examples are provided to illustrate principles of ERT. Another promising approach, namely Schema Therapy (Fassbinder et al.) is presented for its potential to foster emotion regulation in severely disturbed patients (Dadomo et al., 2016). A comparison with Dialectical Behavior Therapy (Linehan, 1993) is made to illustrate different ways the clinician can help patients to regulate emotions. The importance of mindfulness and mindfulness based therapy to produce emotion regulation (Grecucci et al., 2015b) is also explored in this section (Guendelman et al.). The relevance for therapy of motor behavior, with its connection to proprioceptive and interoceptive mechanisms, is also discussed (Shafir). Another paper explores the possibility of improving parenting programs for behavioral disorders in children using the Rational Positive Parenting Program (rPPP), a program with a special focus on parent emotion-regulation functional reappraisal strategies, which has recently received consistent support for reducing child externalizing and internalizing disorder (David et al.). Dingle et al. present evidence of *Tuned In*, a new emotion regulation intervention that uses dedicated music to evoke emotions in session and teaches participants emotional awareness and regulation skills. The special issue ends with reflections on synchrony and psychotherapy, illustrating the Interpersonal Synchrony (In-Sync) model of psychotherapy (Koole and Tschacher). This model considers the alliance between patient and client as grounded in the coupling of the patient's and therapist's brains. Because brains do not interact directly, movement synchrony may help to establish inter-brain coupling.

## Two keys to the future of the field of emotion regulation

This area of research is young, complex, and challenging, but also exciting. While scientific evidence is slowly and partially emerging, no general consensus has been reached yet on how to interpret these early findings. Starting from the papers in this issue, we propose two key questions that scientists and clinicians may want to concentrate on in the near future.

### Cognitive or experiential regulation of emotions?

In the widely accepted Cognitive Emotion Regulation (CER) model of Gross (1998), following classic Appraisal theory, cognitive appraisals of events generate the emotional response. Emotion dysregulation occurs due to the failure to apply appropriate cognitive, attentive, and behavioral regulatory strategies. Cognitive behavioral therapies follow this model, teaching patients to apply behavioral strategies (exposure for example), attentional strategies (increasing attentional flexibility), and cognitive strategies (cognitive restructuring) (see Renna et al. this issue, for an example). An alternative emerging model, known as Experiential-Dynamic Emotion Regulation (EDER) (Grecucci et al., 2015a, 2016a), based on Affective Neuroscience findings (Panksepp, 1998; Damasio, 1999; Panksepp and Biven, 2012; Grecucci and Job, 2015), posits that events trigger emotional responses prewired at birth with inborn adaptive action tendencies. The brain regulates emotions through a biological mechanism. Emotions rise in intensity, peak, and then go flat once the emotion adaptive action tendency has been expressed. The resulting shape of the affective experience is a Gaussian-like shape, proportional to the intensity of the stimulus. This model posits that emotions are not inherently dysregulated. Dysregulation results when emotions are paired with excessive conditioned anxiety, or when affects are triggered by certain defensive strategies, both of which lead to dysregulated-affective states (DASs) (Grecucci et al., 2015a, 2016a). To regulate DAS, the clinician regulates the dysregulating anxiety paired with the emotion or removes defenses which cause the dysregulated affects. These models offer differing conceptualizations for: (1) the generation of emotional response; (2) the causation of dysregulated affects; and (3) the strategies for affect regulation (see Fassbinder et al. this issue, Dadomo et al., 2016; Grecucci et al., 2016b for applications). Research is needed to clarify these mechanisms and how to integrate them. We hypothesize that both processes act as a dual system to foster top-down (cognitive), and bottom-up (experiential) regulation. The clinician can choose moment by moment whether affect regulation would be fostered best by either top-down (cognitive) or bottom up (experiential) strategies.

### What are the basic neurocognitive mechanisms behind the regulation of emotions?

Before the 1990s, neuroscientists agreed that once an emotional learning took place, it was “forever” (LeDoux et al., 1989). The only possible means to “change” that learning was to suppress it with a procedure known as extinction. Extinction offers new learning to decrease the conditioned response when the conditioned stimulus is presented. We hypothesize that cognitive therapies (Renna et al.) rely mainly on extinction to foster emotion regulation through the use of new cognitive strategies. However, extinction based approaches suffer from relapse over time. Luckily, in 1997 evidence of a complete erasure of emotional learning was experimentally provided (Roullet and Sara, 1998; Przybyslawski et al., 1999; Nader et al., 2000; Nader and Einarsson, 2010; Ecker et al., 2013). This process is known as Memory Reconsolidation (Nader et al., 2000; Nader and Einarsson, 2010). The target emotional learning is reactivated in a labile (plastic) state that allows the learning to be erased by offering the experience of an opposite emotional experience (see Ecker et al., 2012 for its clinical applications). We hypothesize that once a Memory Reconsolidation process is reached in the therapeutic setting, the patient can bear the feelings his defenses formerly warded off. Since the defense is no longer necessary, it no longer provokes the dysregulated affects. Likewise, since the patient is able to bear the formerly warded off feelings, they no longer trigger the previous level of anxiety which was dysregulating. As a result, the dysregulated affect and the associated mechanisms that produce the dysregulation cease to exist. Interestingly, different models of therapy (primarily, but not exclusively, experiential approaches) have recently arrived at similar conclusions and implemented similar processes in their practice even before Memory Reconsolidation was discovered. We believe all treatment modalities based on active working and reworking of target emotional learnings (by means of experiential techniques, such as mindfulness (Guendelman et al.), psychodynamic therapy (Rice), and Schema Therapy (Fassbinder et al.) foster Memory Reconsolidation. Research is needed to understand the roles of extinction and memory consolidation in emotion regulation and how to foster them in therapeutic settings.

## Author contributions

AG wrote this editorial, RJ and JF corrected it and added some observations.

### Conflict of interest statement

The authors declare that the research was conducted in the absence of any commercial or financial relationships that could be construed as a potential conflict of interest.
